# Role of mitochondrial genetic interactions in determining adaptation to high altitude human population

**DOI:** 10.1038/s41598-022-05719-5

**Published:** 2022-02-07

**Authors:** Rahul K. Verma, Alena Kalyakulina, Ankit Mishra, Mikhail Ivanchenko, Sarika Jalan

**Affiliations:** 1 Department of Biosciences and Biomedical Engineering, Indian Institute of Technology Indore, Khandwa Road, Simrol, Indore, 453552 India; 2 Department of Applied Mathematics and Centre of Bioinformatics, Lobachevsky State University of Nizhny Novgorod, Nizhny Novgorod, Russia; 3 Complex Systems Lab, Department of Physics, Indian Institute of Technology Indore, Khandwa Road, Simrol, Indore, 453552 India; 4 Laboratory of Systems Medicine of Healthy Aging and Department of Applied Mathematics, Lobachevsky University, Nizhny Novgorod, Russia

**Keywords:** Complex networks, Genomics, Evolution

## Abstract

Physiological and haplogroup studies performed to understand high-altitude adaptation in humans are limited to individual genes and polymorphic sites. Due to stochastic evolutionary forces, the frequency of a polymorphism is affected by changes in the frequency of a near-by polymorphism on the same DNA sample making them connected in terms of evolution. Here, first, we provide a method to model these mitochondrial polymorphisms as “co-mutation networks” for three high-altitude populations, Tibetan, Ethiopian and Andean. Then, by transforming these co-mutation networks into weighted and undirected gene–gene interaction (GGI) networks, we were able to identify functionally enriched genetic interactions of *CYB* and *CO3* genes in Tibetan and Andean populations, while NADH dehydrogenase genes in the Ethiopian population playing a significant role in high altitude adaptation. These co-mutation based genetic networks provide insights into the role of different set of genes in high-altitude adaptation in human sub-populations.

## Introduction

Paramount success of network science banks heavily on the impact of pair-wise interactions among the constituents of complex systems on their evolution and performance. Various complex biological systems and phenomena have been studied through their underlying interaction networks^1^; for example, to infer the role of proteins^2^, to understand biological functions of neurons in various developmental stages of C. elegans^3^, to identify crucial structural patterns in diabetes mellitus II^4^, and to understand genes responsible for the evolution of specific characteristics of human sub-population^5^. Networks allow the modeling of real-world complex systems by a straightforward yet effective framework that consists of nodes and edges. Analyzing the structural and other features of the underlying network properties based on the information available of the edges (interactions) could reveal many system-level properties of corresponding complex systems^6^. The current work provides a model to capture mitochondrial genomic variations by considering them as co-mutation networks and analyze the role of polymorphic cohorts in high altitude (i) Andean Altiplano, (ii) Qinghai–Tibetan Plateau, and (iii) the Ethiopian human population. The mitochondrial genome plays a significant role in determining energy and heat production under specific conditions. It is now believed that about 25% of the mtDNA protein sequence variations^7,8^ 10 to 20% of the tRNA variations, and at least a few of the rRNA variations have altered the mitochondrial coupling efficiency^9^. These alterations led to increased heat production at the expense of ATP production, eventually permitting humans to adapt to colder climates. However, the association and mutual role of polymorphisms in other regions of mtDNA as a complex system subject to investigation in establishing a conclusive role. These three high-altitude populations can be viewed as an outcome of independent replications of a natural experiment of convergent evolution. In such a case, an ancestral founding population moved from low to high altitude, and its descendants have been exposed for millennia to the opportunity for natural selection to improve their functions under high-altitude hypoxia^10^. The term ’convergent evolution’ is defined as the development of the same or similar phenotypic adaptations under a similar external environmental condition as a consequence of natural selection. Although, it could not be denied that the recent multiple migrations from corresponding lower-altitudes could affect the particular signature of high-altitude when compared with the respective low-altitude populations^11^.

These three populations are believed to be evolved differently at the genetic^12^ and physiological levels^19^. Various factors such as genetic pathways, molecular pathways, and phenotype levels have been attributed to the convergent evolution of humans and domestic animals^10^. Studies on the identification of nuclear genes for positive selection in highlander populations have provided evidence for natural selection in the genes responsible for hypoxia-related pathways^13^. In Tibetan highlanders two nuclear genes of the HIF (hypoxia inducible factor) pathway, HIF2A and PHD2 are known to be associated for positive selection^14^. The genetic variations in these genes were also found to be associated with hemoglobin concentration in Tibetans. Additionally, the presence of two non-coding SNPs, rs12097901 (C127S) and rs186996510 (D4E) were found to be as key variations in Tibetan highlanders^15^. Retrospectively, introgression from Denisovan or Denisovan-related individuals has been suggested to be affecting the pattern of high-altitude adaptation in Tibetans^16^. In Andean highlanders, out of 40 genes exhibiting positive selection, the α-1 catalytic subunit of adenosine monophosphate-activated protein kinase (PRKAA1) gene has a significant role in high-altitude adaptation^17^. Further, among Ethiopian highlanders, the positive genetic signatures are known for aryl-hydrocarbon receptor nuclear translocator 2 (ARNT2), basic HLH family member e41 (BHLHE41), vav 3 guanine nucleotide exchange factor (VAV3), mitochondrial calcium uptake 1 (MICU), and thyroid hormone receptor (THRB) genes^18^. Among these three high-altitude populations, Andean and Tibetans represented similar set of genes for positive selection with specific attention to PHD2 gene than the Ethiopian population^18^.

The physiology is higly affected by less oxygen in the inhaled air at high altitudes, results in a lack of oxygen in the bloodstream flowing to the cells for oxygen-requiring energy-producing metabolic reactions in the mitochondria. Based on the factors contributing to arterial oxygen content like hemoglobin content, oxygen saturation, hemoglobin affinity, etc., there exist large pieces of evidences of the Andean-Tibetan difference for high-altitude adaptation. It has already been established that the three high-altitude populations posses significant differences at physiological and genomic levels from their respective low-altitude populations^19,20^. Andeans and Tibetans were reported to show increased hemoglobin concentration compared to corresponding low altitude individuals whereas, Ethiopian high-altitude dwellers reported no significant difference in their hemoglobin level with their low-altitude counterparts^21^. Among these three populations, Andeans showed the highest hemoglobin concentration in their blood. Another physiological trait associated with high altitude is oxygen saturation. Tibetan individuals showed the lowest oxygen saturation, followed by Andeans, and Ethiopians showed oxygen saturation values equivalent to sea level. These findings suggested that Andeans are less stressed by hypoxia than Tibetans, and Ethiopians can provide enough oxygen to their tissues even in a hypoxic environment. In summary, Andean characteristics are high hemoglobin concentration, higher arterial oxygen content, and low oxygen saturation than sea-level reference values. The Tibetans are characterized by sea-level hemoglobin concentration below 4000 m, moderate oxygen saturation, and lower arterial oxygen content than sea-level references values. The Ethiopian patterns of hemoglobin concentration, oxygen saturation, and arterial oxygen content were found to be similar to those of low-altitude dwellers^19^. Apart from physiological differences, there exist specific polymorphisms belonging to mitochondrial genes *ND3* and *CYTB*, which are believed to be associated with high-altitude adaptation in the Tajiks population in Tibet native to China^22^. It has been observed that genetic polymorphisms rarely impart their effect at phenotypic levels individually but as a cohort of multiple interactions together^23^. Single nucleotide polymorphisms (SNPs) have been displayed to have a negligible impact on the heritability of few complex diseases^24^. Additionally, various studies have indicated that interactions of SNPs^25–27^ are one of the critical factors in the manifestation of such complex diseases. Various computational methods have been developed and implemented to select a particular cohort of the variations and their interactions responsible for the manifestation of complex phenotypes. Such methods include principal component analysis to evaluate SNP correlations^28^, integrative scoring system based on their deleterious effects^29^, and Pareto-optimal approach^30^. There exist other approaches based on pair-wise interaction such that two variations significantly interacting through logic regression^31^, predictive rule inference^32^, and shrunken methodology^33^. Networks of variable sites were analyzed to identify genes of angiogenesis associated with breast cancer^34^, time-dependent weight dynamics in chickens^35^, feed efficiency in duroc and landrace pigs^36^, altitude-dependent interactions of mitochondrial genes in Asian population^37^.

In the current work, we analyze the possible role of mitochondrial co-mutations for these high-altitude populations in light of possible convergent evolution, using mtDNA genomes under the framework of networks. Foremost, we constructed the co-mutation network by selecting significantly interacting variations of the mitochondrial genome. These networks were found to follow the small-world behavior with high modularity. The weak ties, nodes with a low degree and high betweenness centrality, were found only in the Tibetan network and acted as haplogroup markers. Followed by that, a gene–gene interaction (GGI) network was constructed from the corresponding co-mutation networks for each population, and functional enrichment analysis was performed based on significantly interacting gene sets. Investigations of GGI networks pointed out essential role of *CYB* and *CO3* genes for high-altitude adaptation in Tibetan and Andean populations while *ND* genes for the Ethiopian population.

## Methods

### Sequence acquisition

Complete human mitochondrial genome sequences were downloaded from the Human mitochondrial Database (HmtDB)^38^ for the Ethiopia and Andes regions situated ∼3000 m, and ∼3500 m above sea level, respectively. For the Andes region, we have downloaded sequences from the Peru region since it inhabits the indigenous Andean (Aymara and Quechua) population. Tibetan sequences (∼4000 m) were downloaded from the GenBank. Accession numbers and references are provided for all the sequences separately as supplementary information. All the sequences were aligned globally and mapped with a master sequence rCRS (revised Cambridge Reference Sequence)^39^.

### Construction of co-mutation network and gene–gene interaction (GGI) network

**Figure 1:**
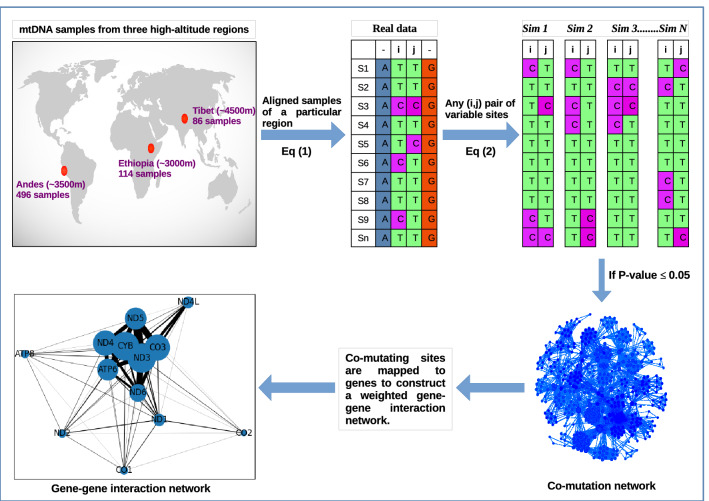
Construction of Co-mutation network and Gene–Gene Interaction (GGI) network for each high-altitude region as explained in “Methods” section.

We constructed two types of networks, first, the co-mutation networks where nodes were variable sites, and second, the weighted GGI networks where nodes were genes (Fig. 1) for each of the high-altitude population.

*Step 1 (co-mutation network)* Any position having more than one allele within the samples is considered a variable site. The variable sites were extracted from the aligned sequences for each region separately. For genomic equality, ambiguous nucleotides such as X, M, Y, etc. were replaced with ‘N’ for all the sequences and tri-allelic sites were not considered.

*Step 2* To construct a network for each high-altitude region, nodes were represented by the position of variable sites, and the edges were represented by co-mutation frequency between a pair of the nodes (*C*_*i j*_) defined, as

1 $${C_{ij} = \frac{{\left( {m_{ij} } \right)^{2} }}{{m_{i} m_{j} }}}$$

where (*m*_*ij*_) represents number of times the minor alleles occur together (defined as co-mutation pair) at *ith* and *jth* positions, *m*_*i*_ and *m*_*j*_ indicate total number of times the minor allele occurs at *ith* and *jth* position, respectively.

*Step 3 (p-value calculation)* To check the significance of any co-mutation pair, the threshold has been calculated as,

2 $${P_{ij} = \frac{{\left[ {\left( {C_{ij}^{r} } \right) \ge \left( {C_{ij} } \right)} \right]}}{{10^{4} }}}$$

where (*C*^*r*^_*ij*_) is the co-mutation frequency calculated after permuting the alleles at the *ith* and *jth* positions randomly. 10,000 random simulations were generated and *Pi j* was set to ≤ 0.05 (standard value) to consider a co-mutation pair significant.

*Step 4 (gene–gene interaction (GGI) network)* Nodes were mapped to their corresponding genes for each co-mutation network to achieve one weighted gene–gene interaction network for each high-altitude region. Since two or more co-mutation pairs may belong to the same gene or a gene pair, each link was counted as many times as it was found, and this number was considered as weight for that gene-pair. For example, the co-mutation pairs (3352–7623) and (4125–8054) would map to *ND1-CO2* gene pairs, so this edge was counted twice, and so on, similarly, the co-mutation pairs (3352–3489) would map to a self-loop for *ND1* gene. Since the variable sites contributed by each gene were in proportion to their lengths (except CR), the weight of a gene-pair was normalized by the sum of the total length of both the genes for that gene-pair. Additionally, we constructed GGI networks using the co-mutations of the (i) common nodes and (ii) exclusive nodes for all three regions. For (i), we took the nodes commonly present in all the networks and scanned for their co-mutating partners, and mapped these co-mutating nodes to respective genes. Similarly, exclusive nodes for each region were used to construct the corresponding GGI network.

### Null model and significant interactions

Random networks were generated corresponding to each co-mutation network to compare with the real networks by taking the same number of the nodes as in the real network, and we connect them with a probability α such that the total number of connections the random networks has the same as of the corresponding real-world;

3 $${\alpha = \frac{{2N_{c} }}{{N\left( {N - 1} \right)}}}$$

where, *Nc* is total number of connections and *N* is total number of nodes in the real network. Further, these random networks were used to get the random GGI networks as described in *Step 4*. We compared the real GGI networks with the corresponding random GGI networks and considered only those pairs significant whose weights fell two standard deviations away from the corresponding random one.

### Detection of community and role of nodes

By calculating the modularity, we detected the communities using the algorithm given in^40^ which is implemented in Python using *community module*. It is a modularity maximization algorithm. The role of each node in the communities is determined by its within-module degree, *Z* score, and the participation coefficient *P*. The within-module degree quantified the node’s intra-modular connectivity and was calculated as the Z-score-transformed degree of centrality within the module. For a given node *i*, *Z*_*i*_ is defined as,

4 $${Z_{i} = \frac{{k_{i} - \overline{k}_{i} }}{\sigma }}$$

where *k*_*i*_ is degree of the *ith* node in its own community, ${\overline{k}_{i} }$ is the average of *k*_*i*_ for all the nodes of that community, and σ is the standard deviation. *Z*_*i*_ takes a high value if degree of *ith* node is high within the cluster and vice versa.

Different roles of a node can also be deduced based on the number of connections the node makes with the nodes in the modules other than its own. For example, two nodes with the same Z-score will play different roles if one of them is connected to several nodes in other modules while the other is not. We define the participation coefficient *P* of node *i* as,

5 $${P_{i} = 1 - \sum\limits_{S = 1}^{Nm} {\left( {\frac{{k_{i} }}{{K_{i} }}} \right)^{2} } }$$

*Ki* is the total degree of the node *i* in the whole network. *S* is the community and *Nm* is the total number of communities. The participation coefficient of a node is therefore close to 1 if its links are uniformly distributed among all the modules and 0 if all its links are within its own module.

### Ethics statement

Ethical review and approval was not required for openly available human database. All methods were performed in accordance with relevant guidelines and regulations.

## Results

### Identification of significant interactions

**Figure 2:**
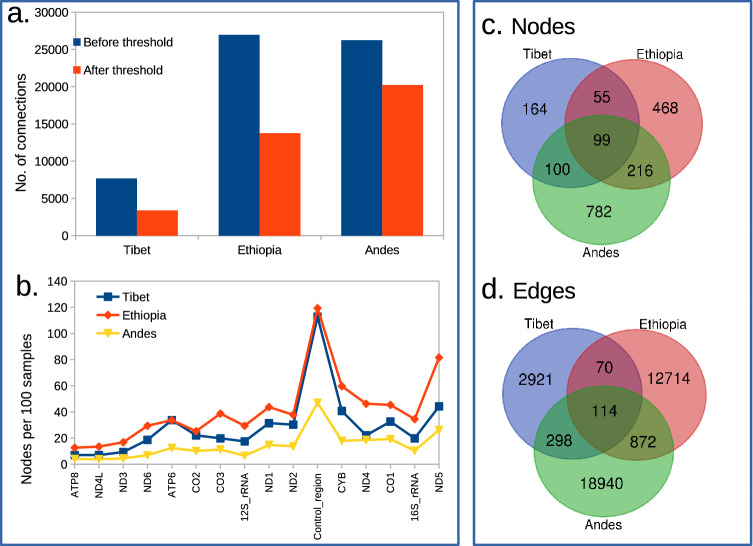
(**a**) The change in number of connections with threshold, (**b**) Nodes which are participating in network construction were mapped to their respective genes and genes were counted and plotted on y-axis with their lengths on x-axis. Note that t-RNA genes are not shown. (**c**) Distribution of nodes and (**d**) co-mutation pairs across all the three regions.

Among the three regions, the Andes region has the highest number of samples, nodes and connections (Table 1). More number of the samples rendered more number of interactions to be statistically significant, which is observed in the comparison of the number of connections (*L*) at *C*_*ij*_ > 0 between the Andes and Ethiopia. Both the regions were having an almost equal number of connections before applying the threshold. When significant pairs (with *P*_*ij*_ ≤ 0.05) were considered, the number of connections were decreased by ~ 23% in Andes, ~ 49% in Ethiopia and ~ 53% in Tibet (Fig. 2a). It was further noted that above the threshold value, the number of co-mutations with low *C*_*ij*_ values (< 0.2) were less, while co-mutations with high *C*_*ij*_ (> 0.2) remained unaffected. This observation signifies the role of *p* value in determining the considerable interactions. Further, the distribution of *C*_*ij*_ for Andes population showed a heterogeneous distribution of the variations within the samples, i.e., there exist fewer co-mutations for *C*_*ij*_ > 0.8, indicating minor alleles in question are not always present in the same samples. The number of nodes and edges for all the three regions are also mentioned in Table 1. To get an overall idea for these three regions, we explored the common nodes and connections among these three co-mutation networks (Fig. 2c,d), to note down similarity and the differences among the underlying networks. It was found that in Tibet 41%, in Ethiopia 55%, and in Andes 65% nodes and ~ 90% connections among all the three regions were exclusively to each region. This suggests the co-evolution of mitochondrial variations pertaining to each geographic region.

**Table 1:** Co-mutation networks: the number of nodes and connections in largest connected component.

Region	Sample size	Nodes	Links
Tibet	86	398	3459
Ethiopia	114	838	13,770
Andes	496	1197	20,224

### Structural properties of co-mutation networks

As we have already established that in these co-mutation networks, a node was a nucleotide position and an edge was co-mutation frequency between any given pair of nucleotide positions. The degree of a node provided information about the frequency of co-mutation of any given variable site with the others. A node with a high degree (hub node) corresponds to a variable site undergoing high co-mutation with many variable sites. Such sites play a crucial role in shaping genome-wide co-evolution pattern of a population in view of multiple migrations and admixture events^7^. The hub node in Tibetan (12308, tRNA-Leu) and Andes (10398, ND3) co-mutation networks were commonly present in all three networks. In contrast, the hub node (4104, ND1) of Ethiopian co-mutation network was present in Andean and absent in the Tibetan network. It is noteworthy that these highest degree nodes were found to be haplogroup markers such that 12308 in Tibetan for K and U haplogroups of N lineage, 4104 in Ethiopian for L0, L1, L2, and L5 haplogroups of L lineage, and 10398 in Andean for multiple haplogroups of L (haplogroup frequency: 95%), M (99.5%) and N (17.1%) lineages. Since humans have migrated to the American continent much after the Eurasian migration, all the mtDNA haplogroups out of Africa descended from either M or N lineages. In the Andes, C and D haplogroups of M lineage contribute to 99% to haplogroup frequency. The revelation of these haplogroup markers as high degree nodes suggested the dominance of specific haplogroup backgrounds for each region’s co-mutation of mtDNA variable sites. This also provides the biological relevance to network construction methodology along with the fundamental nature of co-evolution of haplogroup markers. Among the other high degree nodes, A15301G (*CYB* gene) node was found to be shared among all the three regions and was suggested to be a candidate site for functional analyses, and data association^41^.

Further, all the three networks were found to have small-world property characterized by high clustering coefficient^42^ <*C*> *real*/< *C*> *rand* ≫ 1 and small diameter *Lreal*/*Lrand* ∼ 1 as for many other real-world networks (Table 2)^43,44^. The small-world behavior shown by the brain networks suggests the swift flow of information in minimal steps from one region to another. Similarly, in co-mutation networks, the information of change in allele frequency of a certain nucleotide at one position sweeps to another nucleotide at another position in the same mtDNA sample. Although, for these co-mutation networks, it is a subject of further investigation that whether the two nodes connected through more than one step also share the information of change in allele frequency or not. In terms of co-mutation, this provides evidence for the fixation and inheritance of variations as a single cohort, and intragenic constraints^37^ in the human mitochondrial genome. A high <C> also implies that any given variable site prefers to co-mutate with all the other genes throughout the mitochondrial genome except for tRNA genes.

**Table 2:** Global properties of co-mutation networks.

	Tibet	Ethiopia	Andes
Average degree	17	33	34
Clustering coefficient	0.8	0.7	0.8
Modularity	0.7	0.5	0.4
Average path length	4.3	2.8	2.3
Degree-degree correlation	0.4	0.2	− 0.4

**Figure 3:**
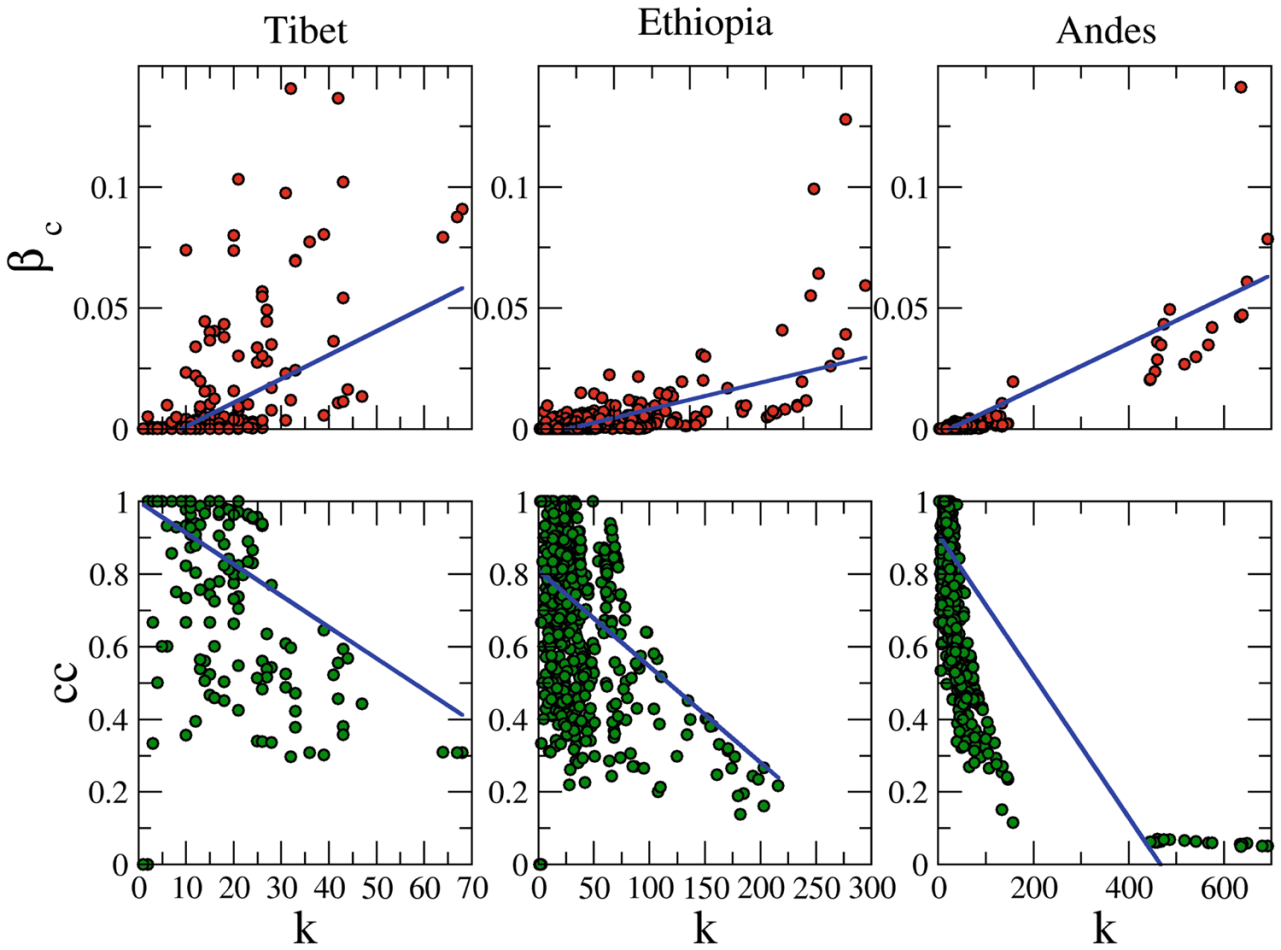
Betweenness centrality (upper panel) and clustering coefficient (lower panel) are plotted as function of degree for all the three regions.

To capture hierarchy, another characteristic property of networks, the clustering coefficient of each node with its degree (Fig. 3, lower panel) was plotted. A decrease in the tendency of a variable site to form clusters with an increase in the degree of that node (Fig. 3) implies the presence of hierarchy^45,46^ in these co-mutation networks. The hierarchical organization confers robustness and adaptability in complex biological networks^47^. mtDNA has acquired several variations depending on biotic and abiotic factors since humans have first migrated outside Africa throughout the world^48^. These enriched variations gave rise to multiple haplogroups. The presence of high clustering and hierarchy in these co-mutation networks might help capture this temporal and spatial co-evolution of variable sites of the mitochondrial genome in the form of haplogroups.

Resilience is an important property for a network, which is measured by the betweenness centrality (β*c*)^6^. This centrality measure estimates the number of shortest paths between any given pair of nodes which increases if a node is removed. Usually, the nodes with a high degree tend to have high betweenness centrality. However, it was observed here that a few nodes, despite having a low degree, have high betweenness centrality and are considered as weak ties. Weak ties are the nodes that co-mutate with a few nodes but from different modules. Presence of weak ties suggests that mtDNA has evolved through co-evolution of few nodes (pertaining to low *k*) of multiple discrete modules (pertaining to high β*c*)^49^. Thus, these sites are significantly important since their removal can result in the breakdown of the network. In the Tibetan co-mutation network, we found four such variable sites (709, 15,927, 16,172, and 16,362). Interestingly, all these nodes were again found to be haplogroup markers (709: L6, G, T, and W; 15,927: G, B, and X: 16,172: L0 and F; 16,362: L4, D, G, and A). This suggests that haplogroup markers provide the necessary evolutionary background and play a key role in assisting the co-evolution of different clusters. Moreover, 15,927 node belongs to *tRNA-Thr* which is one of the highly mutated tRNAs among all the tRNAs in humans^50^, and similarly, variable sites 709 (*12S-rRNA*) in *rRNA*. tRNAs and rRNAs play a central role in protein synthesis. This signifies that tRNAs and rRNAs might play decisive roles in the co-evolution of different mutational cohorts in the Tibetan population. On the contrary, we did not find any such nodes in Ethiopian or Andean co-mutation networks. In these two networks, the nodes with high betweenness centrality also possessed a large degree. This suggests that in Ethiopian and Andean populations, the mtDNA has evolved through continuous co-evolution of many different nodes (of high *k*) of multiple modules (of high β*c*) altogether.

Another characteristic property, the network diameter, defined as the longest of all shortest paths, was large in Tibet compared to Ethiopia and Andes. This large network diameter gives evidence about long-range co-mutation and high modularity in the Tibetan population. To detect modularity in these co-mutation networks we applied the Louvain community detection algorithm^40^ implemented in the python *community module*. The high modularity indicates the formation of mutational cohorts of evolutionary constraints at the whole-genome level. We analyzed the genetic background of the communities formed in these co-mutation networks. On considering only the coding regions, it was observed that nodes of a few particular genes contribute more than other genes in each community in all the three co-mutation networks (Supplementary Table 1). Particularly, in Tibet, *ATP6*, *CYB*, *ND5* genes, in Ethiopia *ND5* gene, and in Andes *ND5*, and *CYB* genes showed considerable contribution among all the communities. *CO1* and *ND2* genes were also found to dominate at least in one community in each of the three populations. We also analyzed the highest degree nodes for the coding region in each community. These high degree nodes were considered "community cores". We found that none of these community cores are common among the three regions. Although the three regions have a certain number of common nodes (Fig. 2), the community structures are derived by independent nodes. This supports the fundamental nature of formation of various haplogroups due to migration patterns and events of natural selection which were derived by a few specific variations^51^. Upon individual inspection of the communities, we found that in each of the three regions, despite contributing few nodes *tRNA-Leu, tRNA-Lys, and tRNA-Gln* were found to be community cores in the Tibet, Ethiopia, and Andes regions, respectively. Apart from that, the *CR* was found to be evenly present in all the communities.

We investigated the localization properties of eigenvectors of these co-mutation networks. Localization of eigenvectors enjoy a wide range of applications in networks; in disease spreading^52^, perturbation of propagation in mutualistic networks^53^. Other applications of localization can also be found in^54,55^. To quantify localization, we used correlation dimension (*D*2) calculated by using the box-counting method for multifractal analysis of eigenvectors^56^. If *D*2 → 0, eigenvector is said to be localized while if *D*2 → 1, eigenvector is considered as delocalized. Thus, *D*2 provides insight into the degree of localization of eigenvectors. We focus on the eigenvectors of eigenvalues nearer to zero and *D*2 is averaged over all the eigenvectors inside width *d*λ = 0.5. Note that, slight increase or decrease in the width will not alter the results. Tibetan and Andean networks were more localized, with *D*2 being 0.43 compared to the Ethiopian network with *D*2 0.65. In these networks, the tendency of co-mutation is captured by *D*2 in terms of localization. A co-mutation occurs when minor alleles at any given two sites are present in considerable frequency in the population. The change in this co-mutation frequency is further affected by the introduction of new DNA samples harboring that particular minor allele. In other words the co-mutation is being localized around a few sites. Migration and natural selection are few of the events which might cause a change in allele frequency at certain positions, which further affects the tendency of that site to co-mutate. In Andean and Tibetan populations, the co-mutation has been localized compared to Ethiopia. This might be possible due to the recent population admixture experienced by the Ethiopian population^11^ due to its comparatively lesser harsh environment than Tibet and Andes. Thus, population admixture might have played a role in observed localization behavior in these three populations.

**Figure 4:**
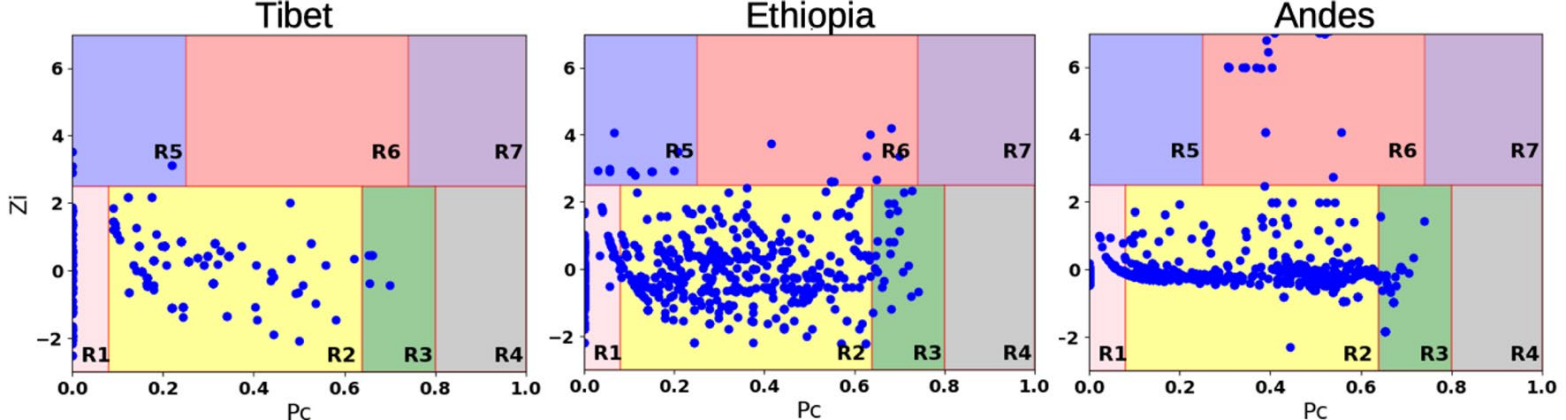
Roles of nodes in Z–P parameter space. Each node in a network can be characterized by its within-module degree and its participation coefficient. Nodes with Z 2.5 were classified as module hubs and nodes with Z < 2.5 as non-hubs. Non-hub nodes can be naturally assigned into four different roles: (R1) ultra-peripheral nodes; (R2) peripheral nodes; (R3)non-hub connector nodes; and (R4) non-hub kinless nodes. Hub nodes can be naturally assigned into three different roles: (R5)provincial hubs; (R6) connector hubs; and (R7) kinless hubs.

Further, for these networks, there exists no node with its degree distinctly very high than those of the other nodes, and hence the importance of a node cannot be assigned based on its degree only. Nevertheless, due to the presence of the high modularity, the importance of a node can be determined, to a great extent, by its within-module degree and participation coefficient, which defines how a node is positioned in its own module and with respect to other modules^57,58^. Based on the within-module degree and the participation coefficient, nodes were categorized as module hubs and non-hubs (Fig. 4). The highest degree nodes for the Tibetan co-mutation network were found in R3, non-hub connector category, while for the Ethiopian and Andean co-mutation networks, the highest degree nodes were found in the R6 connector hubs category. In the Tibetan co-mutation network, the nodes in R5 category were 3010 (*16S_rRNA*), 8414 (*ATP8*), 14,668 (*ND6*) and 12,361 (*ND5*). Variable site 3010 was shown to be a high-altitude marker in Tibetan population^59^ and also reported from network motifs with variable sites 8414 and 14668^37^ while, 12,361 was shown to be associated with nonalcoholic fatty liver disease^60^. It is noteworthy that in the Tibetan co-mutation network, there were no nodes in the R6 category, and in the Andean co-mutation network, there were no nodes in the R5 category, while in the Ethiopian co-mutation network nodes were present in both the R5 and R6 categories. As we know, provincial hubs (R5) tend to connect nodes within the same module while connector hubs (R6) tend to connect nodes from different modules. Based on the observation that the modules inherit the information of haplogroups, we can consider R5 nodes as intra-module hubs and R6 category as inter-module hubs. It can be inferred that in Tibetan population, intra-haplogroup co-evolution is prominent, while in the Andes, mtDNA inter-haplogroup co-evolution is prominent. On the other hand, Ethiopian mtDNA harbors both inter and intra-haplogroup co-evolution. This again provides evidence for recent admixture in Ethiopian region^11^. These co-mutation networks are then analyzed at the gene level through the gene–gene interaction networks, discussed further.

### Gene–gene interaction (GGI) networks

The contribution of each gene was quantified by counting the number of variable sites from each gene (Fig. 2b). It was observed that the number of nodes in the network were proportional to the length of genes, hence we normalized the number of variable sites with the corresponding gene lengths (Fig. 2b). It was deduced that except for the Control region (CR), the occurrence of variable sites for each gene increased with an increase in the length of genes. *CR* is a mutational hot-spot in mtDNA, hence contributed the highest number of variables sites. Since *CR* does not code for any protein, we did not consider its interaction at the gene–gene network level to avoid bias due to the high mutation rate. It was evident that certain genes contributed more variable sites in the network construction than others in a particular region. Especially, *ATP6, CO2* and *ND2* genes are contributing equally in the Tibetan and Ethiopian networks while *12S-rRNA, 16S-rRNA, CO3* and *ND4* were contributing equally in the Tibetan and Andean networks. Among the coding genes, the ND5 gene showed the highest difference of contributing nodes with the minimum in Tibet and maximum in the Andes (Fig. 2b). The contribution of each gene per 100 samples for the network construction was highest in Ethiopia among all the regions. The nodes pertaining to *CR* displayed the largest participation in the network construction due to the fact that it is the highly variable part of mtDNA^61^. Contribution of the variable sites in each gene yields partial information about the interaction of the genes. To overcome this, we generated the gene–gene interaction networks by mapping the variable sites with the respective genes, as discussed in the *Methods section*. The gene–gene interaction networks provide a holistic and a reductionist approach to investigate interactions in the three high-altitude regions. We found 17 gene pairs in Tibetan, 23 gene pairs in Ethiopian, and 44 gene pairs in the Andes population after comparing with their corresponding random networks. Among these, the pair with the highest edge weight *ATP6-CYB* (Tibet), *ND4-ND5* (Ethiopia), and *CYB-ND4* (Andes). Four significant gene–gene pairs were found to be commonly present in all the three populations, which were *CO1-CO2, ND2-ND4, ND3-ND4* and *ND4-ND5*. All these genes are involved in the oxidative phosphorylation pathway (KEGG entry: 00190) and thermogenesis pathway (KEGG entry: 04714)^62^, along with that these genes are also found to be involved in cellular respiration (GO:0045333), and response to abiotic stimulus (GO:0009628)^63^. At higher-altitudes where low temperature and hypoxia are two main abiotic factors responsible for natural selection, the genes involved in thermogenesis and response to abiotic factors play an imperative role in determining the evolution, and adaptation^20^.

**Figure 5:**
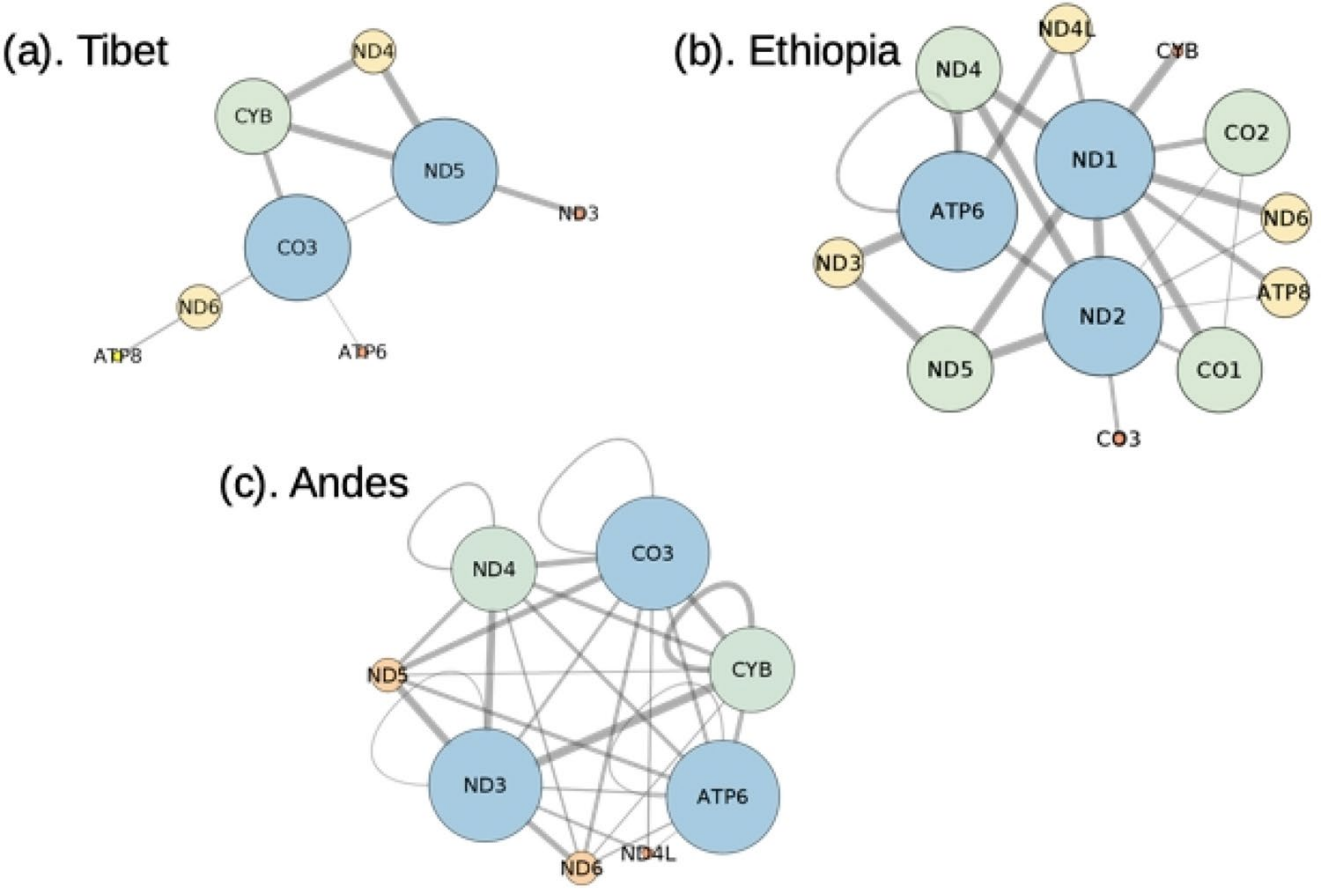
Significant gene–gene interactions of common node co-mutations (the node size depicts the degree of the node and edge size represents the edge weight).

Since these three populations are believed to share a similar physical environment and to undergo the process of convergent evolution, for all the common nodes, we extracted their co-mutations and constructed corresponding GGI. The common variable sites categorized these three populations under the same haplogroups, while their co-mutations differ among these three populations. To capture this difference at the genetic level, significant genetic interaction (Fig. 5) of the common nodes were extracted based on the functional enrichment analysis for GO terms and KEGG pathways using DAVID^64^. It was found that in the Tibetan population *CO3, CYB* and *ND5* genes, in the Andean population *ATP6, CO3, CYB, ND3* and *ND4* genes, and in the Ethiopian population *ATP6, CO1, CO2, ND1, ND2, ND4* and *ND5* genes were significantly interacting with other genes. The functional enrichment of these gene sets is shown in Table 3. It is noteworthy that from the cytochrome oxidase complex, the *CO3* sub-unit was interacting in the Tibetan and Andean populations while *CO1* and *CO2* sub-units were interacting in the Ethiopian population. *CO3* sub-unit is the putative site for the entry of oxygen into the large cytochrome oxidase complex, thereby regulating its activity under hypoxic conditions^65^. Even though the Ethiopian gene set has not shown any feature related to the hypoxia adaptation in functional enrichment analysis, variations in *ND1* and *ND2* genes were shown to be associated with high-altitude hypoxia in Tibetan yak^66^ and endemic Ethiopian rats^67^. Apart from the functional enrichment, the Tibetan and Andean gene-sets were also involved in non-alcoholic fatty liver disease (NAFLD) pathways. It has been shown that high altitude might improve the mitochondrion function and alleviate the NAFLD^68^.

**Table 3:** The GO terms and KEGG pathways for gene sets described in Fig. 5.

	Tibet (CO3, CYB, ND5)	Ethiopia (ATP6, CO1, CO2, ND1, ND2, ND4, ND5)	Andes (ATP6, CO3, CYB, ND3, ND4)
Response to hypoxia [GO: 0001666]	Yes	No	Yes
Response to hyperoxia [GO: 055093]	No	No	Yes
Respiratory electron transport [GO: 022904]	Yes	No	Yes
ATP synthesis coupled electron transport [GO: 042773]	No	Yes	No
Non-alcoholic fatty acid liver disease [KEGG: 04932]	Yes	No	Yes

**Figure 6:**
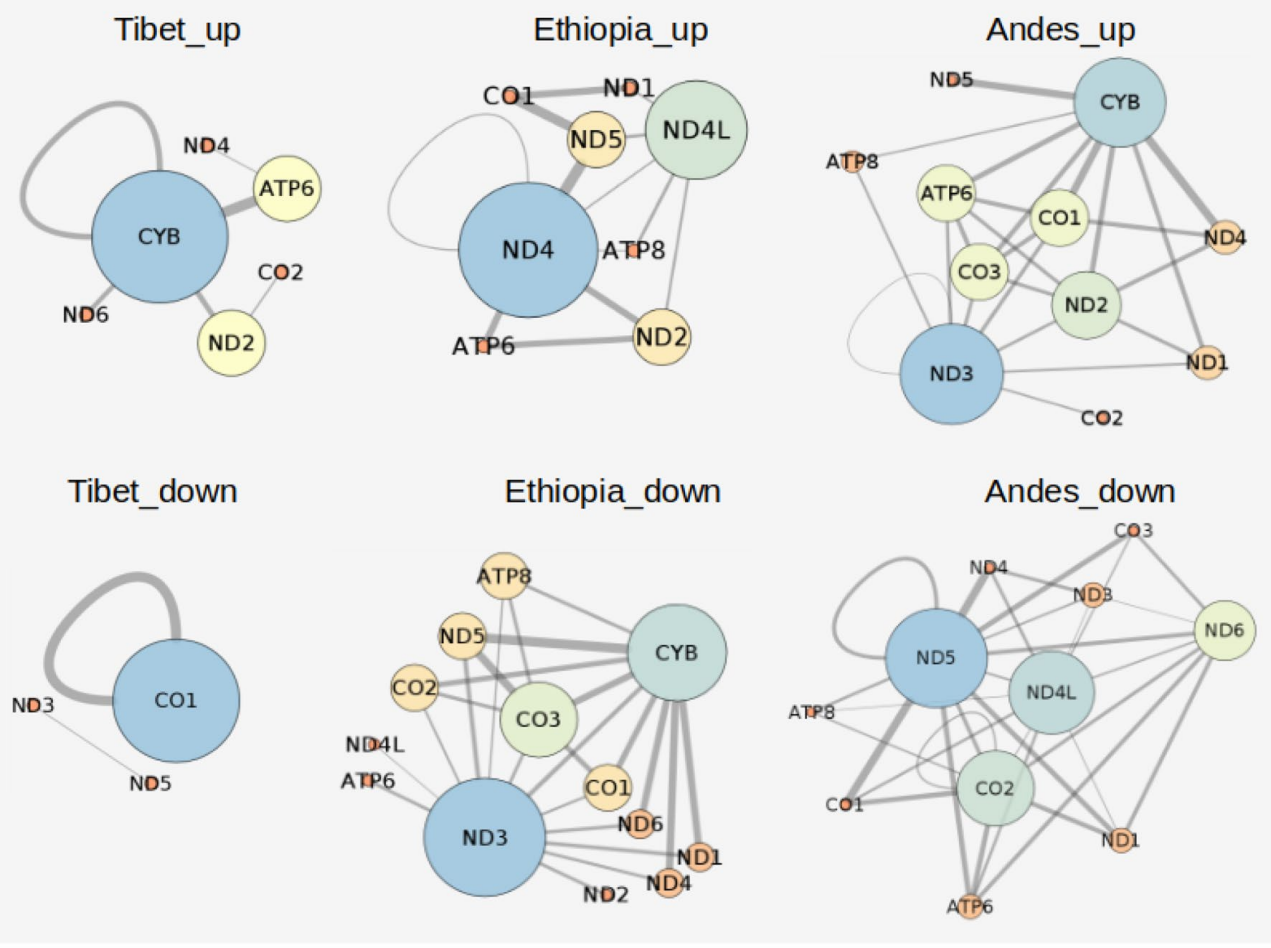
Significant gene–gene interactions of exclusive nodes co-mutations. The Region_up and Region_down represents those interactions which were showing higher and lower weight than the corresponding random networks for each region, respectively (the node size depicts the degree of the node and edge size represents the edge weight).

Further to explore the role of such genetic interactions at the region-specific level, we extracted the co-mutation pairs pertaining to the exclusive nodes of each region and constructed GGI networks. In GGI networks with these exclusive nodes, we found specific interactions with significantly lower weights and others with significantly higher weights than the corresponding random ones (Fig. 6). It is readily observed from Fig. 6 that those genetic interactions that were significantly up in the Andean and Tibetan populations were significantly down in the Ethiopian population and vice versa. This suggests that both Tibetan and Andean populations have evolved at high altitudes through the interactions of *CYB* and *CO3* genes. In contrast, the Ethiopian population deviated in sharing the mitochondrial genetic interactions with the other two populations. This dissimilarity could be explained based on two facts; one is that Ethiopia is situated at the lowest altitude among all the three populations, and the second is that Ethiopia is believed to underwent frequent admixtures in its gene pool from a lower altitude populations^69^.

## Conclusion

Although haplogroups or specific mutations help us categorizing the human population geographically, the proposed co-mutation networks fortify the specific genetic interactions even in similar environmental backgrounds. Our analysis showed that mtDNA has evolved with similar biological mechanisms in Andean and Tibetan populations than the Ethiopian population. We found a heterogeneous set of genes in Ethiopian population than the Tibetan and Andean populations compared to corresponding random networks. Notably, CYB and CO3 genes are commonly present in Tibetan and Andean population, and are interacting with ND5 gene in Tibetan and ATP6, ND3 and ND4 genes in Andean populations. Whereas, in the Ethiopian population four NADH dehydrogenase genes (ND1, ND2, ND4, ND5) showed interactions with ATP6, CO1 and CO2 genes. It was noticeable that in exclusive GGI networks, Ethiopian population showed the contrasting behavior compared to the Tibetan and Andean population. Further, the D_2_ analysis also showed that Tibetan and Andean populations are similar in their localization behavior compared to Ethiopian population. The tendency of variable sites to co-mutate could be affected by introduction of either new samples or new variations in the existing samples, the D_2_ analysis would be employed to capture such admixture or multiple migration events or genetic drift in a particular population. To conclude, co-mutation based genetic interaction networks identified the gene sets which could have played critical role in establishing the human lineage and acclimatization to higher altitudes around the globe. These gene sets and pertaining variable sites provide a ground for further investigation of patterns of human migration and settlements across these three regions.

## Supplementary Information

Supplementary Information 1.

Supplementary Information 2.

Supplementary Information 3.

Supplementary Information 4.

Supplementary Information 5.
