# Supplementary figures and images for: Role of mitochondrial genetic interactions in determining adaptation to high altitude human population
Source: Sci Rep. 2022 Feb 7;12:2046. doi: 10.1038/s41598-022-05719-5 (PMC8821606; doi:10.1038/s41598-022-05719-5)

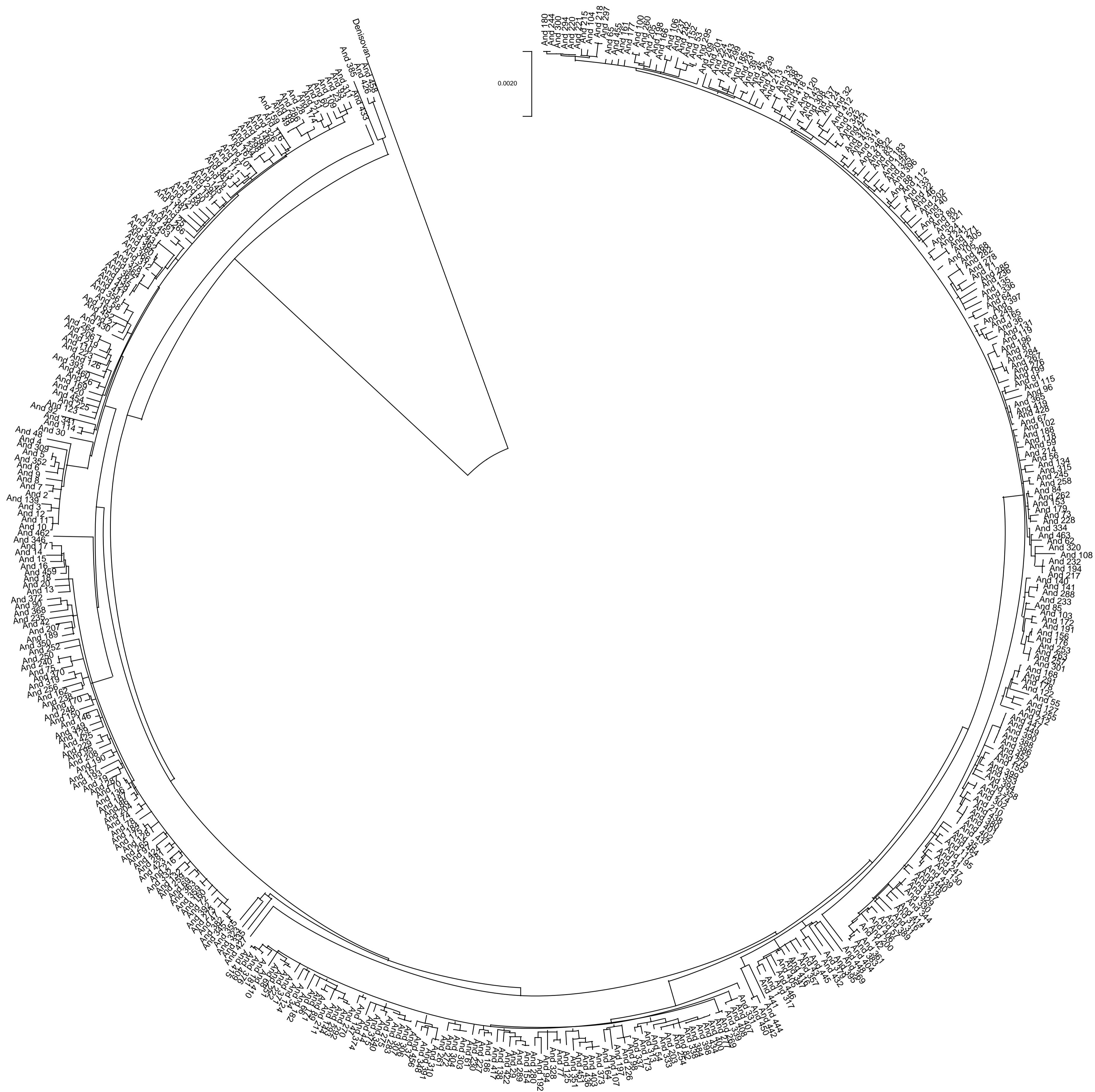

Supplement: Supplementary file 1 — Supplementary Information 1. [file 41598_2022_5719_MOESM1_ESM.pdf]

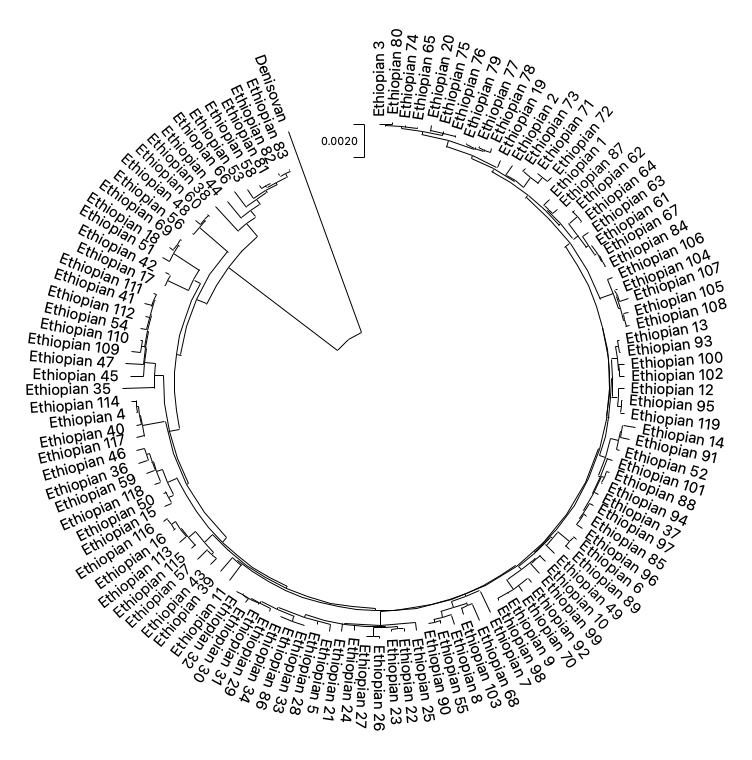

Supplement: Supplementary file 2 — Supplementary Information 2. [file 41598_2022_5719_MOESM2_ESM.png]

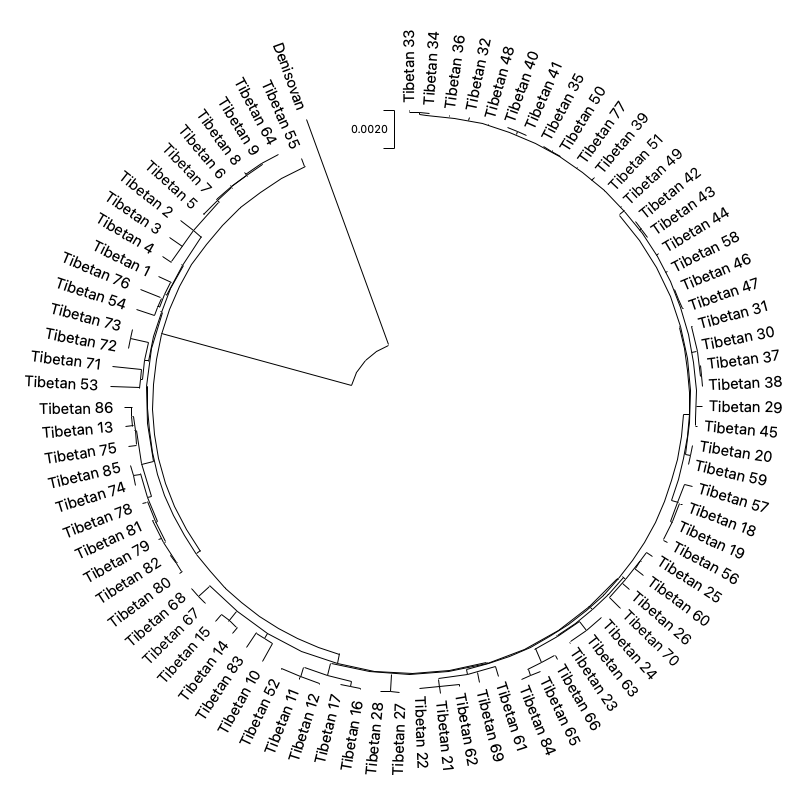

Supplement: Supplementary file 3 — Supplementary Information 3. [file 41598_2022_5719_MOESM3_ESM.png]
