# Supplementary material for: Role of mitochondrial genetic interactions in determining adaptation to high altitude human population
Source: Sci Rep. 2022 Feb 7;12:2046. doi: 10.1038/s41598-022-05719-5 (PMC8821606; doi:10.1038/s41598-022-05719-5)
Supplement: Supplementary file 5 — Supplementary Information 5. [file 41598_2022_5719_MOESM5_ESM.doc]

**Role of mitochondrial genetic interactions in determining adaptation to high altitude in human population around the globe**

**Rahul K Verma**1**, Alena Kalyakulina**2**, Ankit Mishra**3**, Mikhail Ivanchenko**2,4**, and Sarika Jalan**1,3*

1Discipline of Biosciences and Biomedical Engineering, Indian Institute of Technology Indore, Khandwa Road, Simrol, Indore-453552, India
2Department of Applied Mathematics and Centre of Bioinformatics, Lobachevsky State University of Nizhny Novgorod, Nizhny Novgorod, Russia

3Complex Systems Lab, Discipline of Physics, Indian Institute of Technology Indore, Khandwa Road, Simrol, Indore-453552, India
4Laboratory of Systems Medicine of Healthy Aging and Department of Applied Mathematics, Lobachevsky University, Nizhny Novgorod, Russia

*sarika@iiti.ac.in


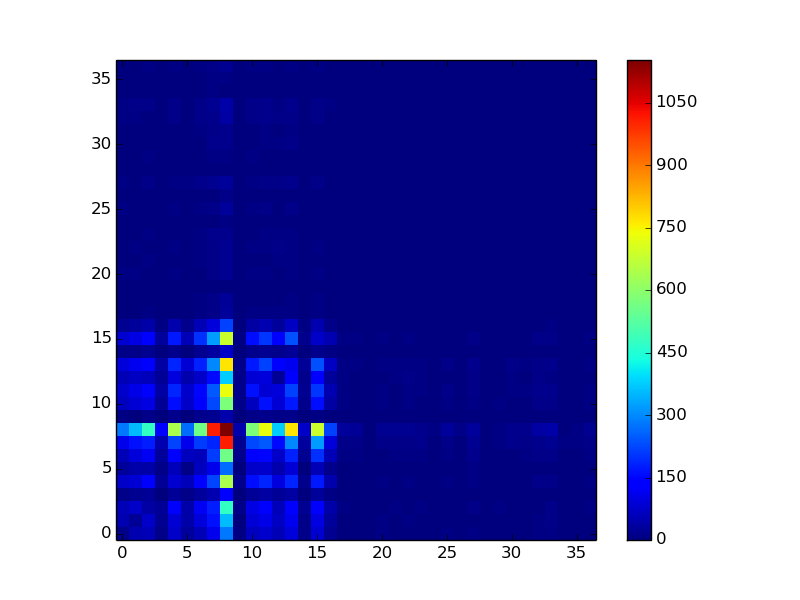


Genes

Genes

Control region

Control region

Andes

Figure S1. (a) Andes: Gene-gene interaction weights are plotted as heat map for all the gene-pairs including Control region. Control region showed highest weights with all the other genes.


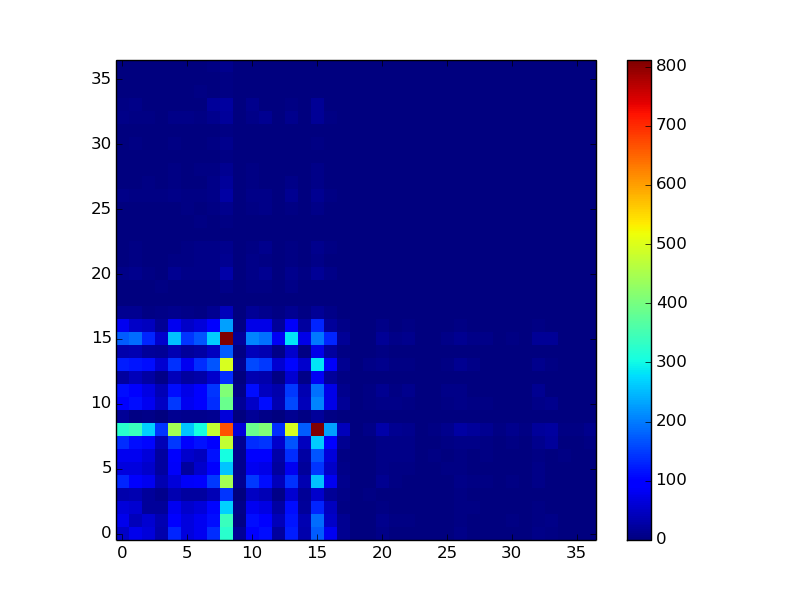


Genes

Genes

Control region

Control region

Ethiopia

Figure S1. (b) Ethiopia: Gene-gene interaction weights are plotted as heat map for all the gene-pairs including Control region. Although, ND5 gene showed highest self-loop weight, Control region showed higher weights with all the other genes.


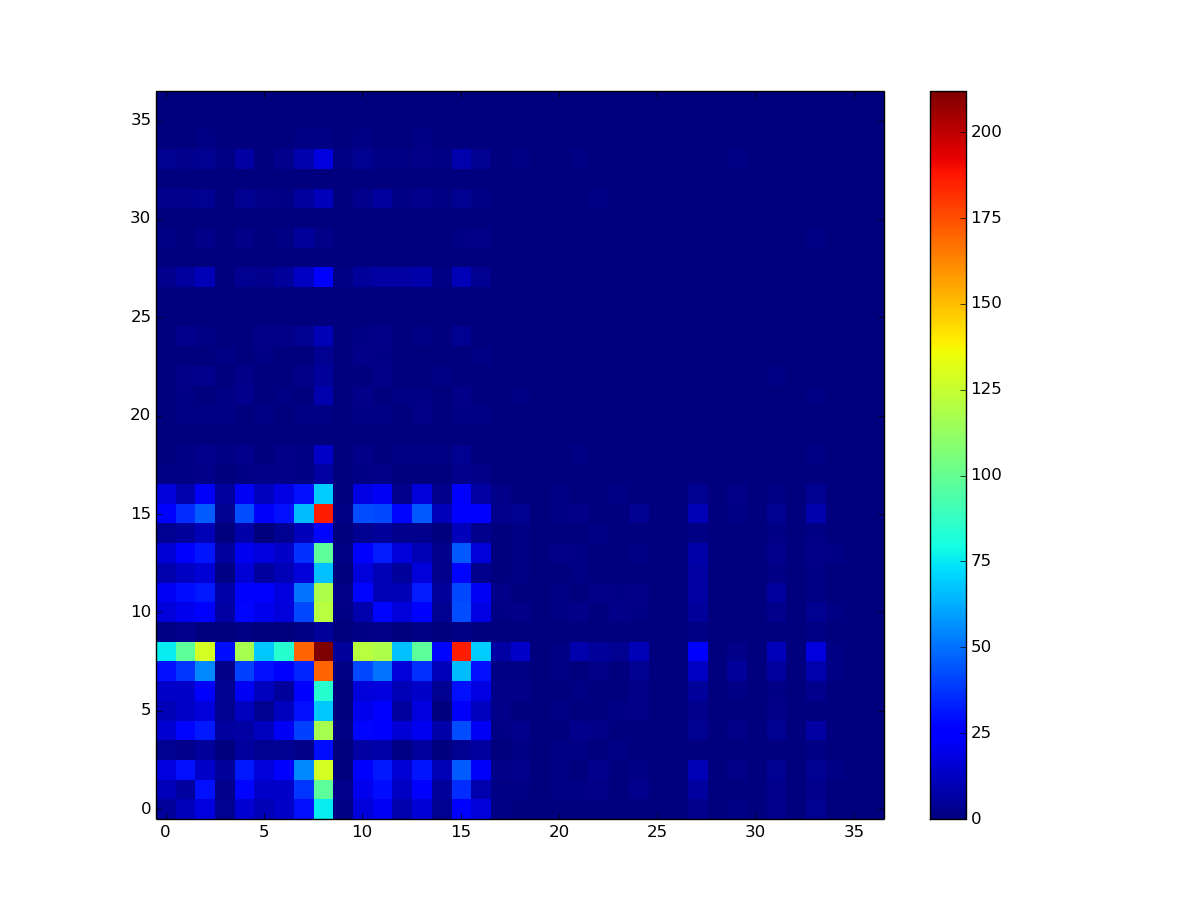


Genes

Genes

Control region

Control region

Tibet

Figure S1. (c) Tibet: Gene-gene interaction weights are plotted as heat map for all the gene-pairs including Control region. Control region showed highest weights with all the other genes. ND5 gene showed higher self-loop weight.

Figure S2. The importance of thresholding is shown as a consequence of co-mutation frequency distribution for all the three regions: (a) Andes, (b) Ethiopia, and (c). Tibet. When thresholding is applied, the connections with lower cf are filtered out hence we are left with statistically significantly connections only. Note the scale at y-axis is changing for with and without thresholding.

a.


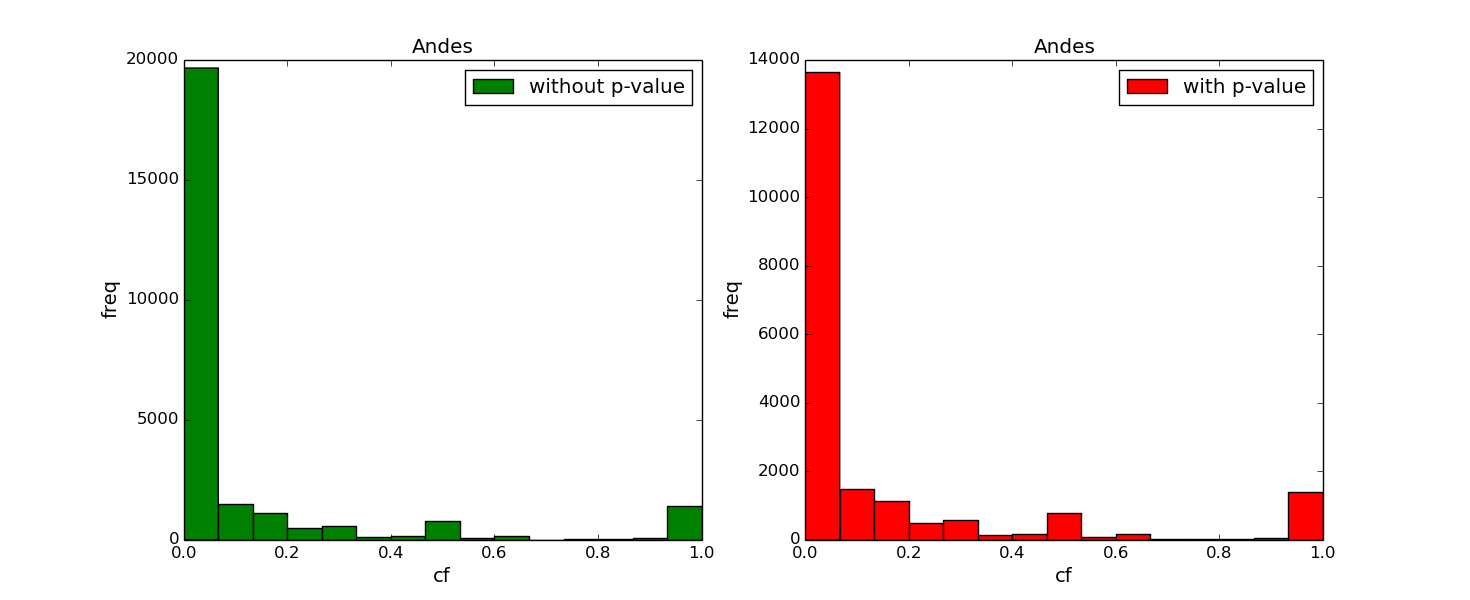


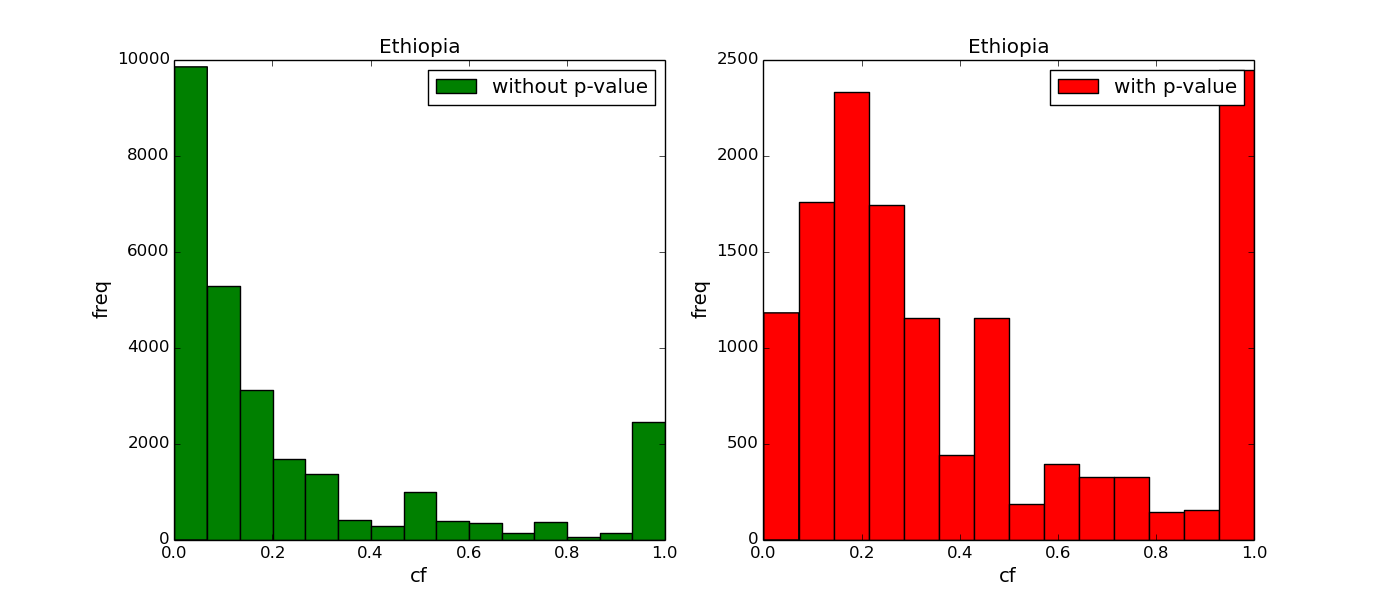


b.

c.


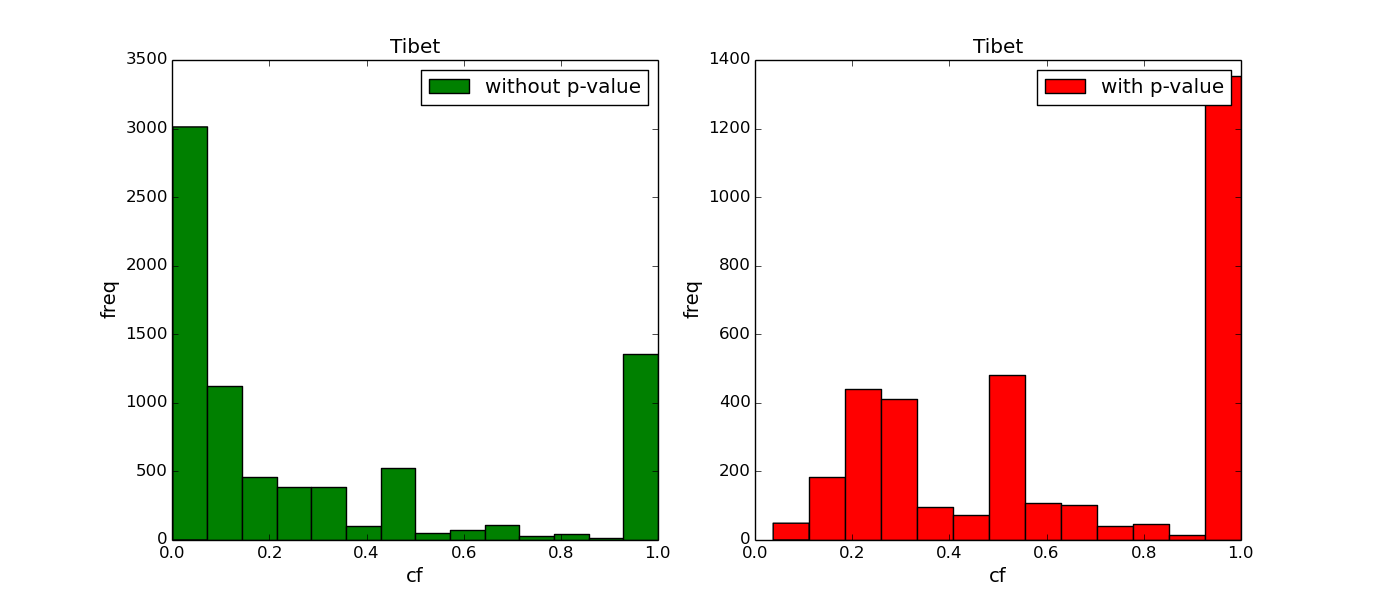


**Figure S3. Phylogenetic trees:**

All the phylogentic trees were generated by Maximum Likelihood method and Tamura-Nei model [1] keeping default parameters using MEGA X[2,3]

References:

1. Tamura K. and Nei M. (1993). Estimation of the number of nucleotide substitutions in the control region of mitochondrial DNA in humans and chimpanzees. Molecular Biology and Evolution 10:512-526.

2. Kumar S., Stecher G., Li M., Knyaz C., and Tamura K. (2018). MEGA X: Molecular Evolutionary Genetics Analysis across computing platforms. Molecular Biology and Evolution 35:1547-1549.

3. Stecher G., Tamura K., and Kumar S. (2020). Molecular Evolutionary Genetics Analysis (MEGA) for macOS. Molecular Biology and Evolution. (https://doi.org/10.1093/molbev/msz312).

**Supplementary Figure Legends**

**Figure S1:** Heatmaps for weighted gene-gene interactions for all the mitochondrial genes specifically mentioned for Control region. All the genes are arranged alphabetically. (a) Andes, (b) Ethiopia, (c) Tibet. These heatmaps were generated in Python (version 2.7) using *matplotlib* (version 1.3.1) library.

**Figure S2:** The importance of thresholding is shown as a consequence of co-mutation frequency distribution for all the three regions: (a) Andes, (b) Ethiopia, and (c). Tibet. When thresholding is applied, the connections with lower cf are filtered out hence we are left with statistically significantly connections only. Note the scale at y-axis is changing for with and without thresholding.

**Figure S3:** The phylogenetic trees were constructed for all the populations along with a Denisovan mtDNA by Maximum Likelihood method and Tamura-Nei model keeping default parameters using MEGA version X (Kumar, Stecher, Li, Knyaz, and Tamura 2018)(Stecher, Tamura, and Kumar 2020). (a) Andes, (b) Ethiopia, (c) Tibet.

**S Table 1***:* Below are the three tables (for each region) showing the percentage of nodes (co-mutation network) belonging to each gene in each community. Size is the number of variable sites in each community. Genes dominating each community are colored blue.

(a). Tibetan

| Sr. no | size | ATP6 | ATP8 | CO1 | CO2 | CO3 | CYB | ND1 | ND2 | ND3 | ND4 | ND4L | ND5 | ND6 | 12s | 16s | trna |
| --- | --- | --- | --- | --- | --- | --- | --- | --- | --- | --- | --- | --- | --- | --- | --- | --- | --- |
| 1 | 65 | 6.2 | 0.0 | 6.2 | 1.5 | 4.6 | 4.6 | 6.2 | 3.1 | 6.2 | 4.6 | 1.5 | 12.3 | 1.5 | 4.6 | 4.6 | 3.1 |
| 2 | 55 | 7.3 | 1.8 | 9.1 | 5.5 | 5.5 | 3.6 | 5.5 | 7.3 | 1.8 | 5.5 | 0.0 | 10.9 | 1.8 | 3.6 | 5.5 | 3.6 |
| 3 | 51 | 0.0 | 3.9 | 7.8 | 7.8 | 2.0 | 2.0 | 9.8 | 2.0 | 2.0 | 3.9 | 0.0 | 5.9 | 3.9 | 0.0 | 3.9 | 11.8 |
| 4 | 52 | 7.7 | 1.9 | 5.8 | 1.9 | 3.8 | 13.5 | 3.8 | 5.8 | 1.9 | 5.8 | 0.0 | 5.8 | 7.7 | 7.7 | 0.0 | 5.8 |
| 5 | 49 | 2.0 | 2.0 | 4.1 | 4.1 | 4.1 | 14.3 | 4.1 | 10.2 | 0.0 | 4.1 | 2.0 | 10.2 | 4.1 | 4.1 | 6.1 | 2.0 |
| 6 | 48 | 12.5 | 2.1 | 6.3 | 2.1 | 4.2 | 8.3 | 4.2 | 4.2 | 0.0 | 2.1 | 4.2 | 8.3 | 8.3 | 4.2 | 0.0 | 6.3 |
| 7 | 38 | 5.3 | 0.0 | 10.5 | 2.6 | 2.6 | 10.5 | 5.3 | 7.9 | 0.0 | 5.3 | 2.6 | 10.5 | 2.6 | 2.6 | 10.5 | 7.9 |
| 8 | 25 | 12.0 | 0.0 | 4.0 | 8.0 | 0.0 | 12.0 | 16.0 | 8.0 | 4.0 | 8.0 | 0.0 | 4.0 | 0.0 | 4.0 | 8.0 | 0.0 |
| 9 | 14 | 7.1 | 0.0 | 7.1 | 7.1 | 14.3 | 0.0 | 7.1 | 14.3 | 0.0 | 0.0 | 0.0 | 7.1 | 7.1 | 0.0 | 0.0 | 7.1 |

(b). Ethiopian

| Sr. no | Size | ATP6 | ATP8 | CO1 | CO2 | CO3 | CYB | ND1 | ND2 | ND3 | ND4 | ND4L | ND5 | ND6 | 12s | 16s | trna |
| --- | --- | --- | --- | --- | --- | --- | --- | --- | --- | --- | --- | --- | --- | --- | --- | --- | --- |
| 1 | 293 | 5.5 | 1.0 | 5.8 | 3.1 | 3.8 | 10.9 | 6.5 | 3.4 | 2.4 | 3.8 | 2.4 | 12.6 | 3.8 | 3.4 | 4.1 | 3.4 |
| 2 | 146 | 4.1 | 2.1 | 7.5 | 4.8 | 3.4 | 4.1 | 6.8 | 6.8 | 2.1 | 5.5 | 2.7 | 11.6 | 4.1 | 5.5 | 7.5 | 3.4 |
| 3 | 127 | 4.7 | 3.1 | 3.1 | 1.6 | 11.8 | 7.1 | 7.1 | 7.1 | 1.6 | 7.1 | 0.0 | 9.4 | 3.9 | 1.6 | 5.5 | 11.8 |
| 4 | 95 | 5.3 | 3.2 | 7.4 | 4.2 | 4.2 | 7.4 | 5.3 | 3.2 | 4.2 | 13.7 | 3.2 | 11.6 | 4.2 | 5.3 | 2.1 | 3.2 |
| 5 | 62 | 3.2 | 0.0 | 8.1 | 9.7 | 4.8 | 8.1 | 4.8 | 8.1 | 0.0 | 9.7 | 3.2 | 12.9 | 9.7 | 3.2 | 3.2 | 3.2 |
| 6 | 38 | 10.5 | 0.0 | 10.5 | 0.0 | 5.3 | 15.8 | 2.6 | 7.9 | 2.6 | 5.3 | 0.0 | 10.5 | 2.6 | 13.2 | 2.6 | 0.0 |
| 7 | 34 | 0.0 | 0.0 | 5.9 | 0.0 | 11.8 | 8.8 | 8.8 | 5.9 | 5.9 | 14.7 | 0.0 | 14.7 | 2.9 | 2.9 | 5.9 | 0.0 |
| 8 | 24 | 4.2 | 8.3 | 4.2 | 8.3 | 0.0 | 8.3 | 4.2 | 12.5 | 0.0 | 4.2 | 0.0 | 8.3 | 0.0 | 4.2 | 12.5 | 12.5 |
| 9 | 5 | 0.0 | 0.0 | 20.0 | 0.0 | 0.0 | 20.0 | 20.0 | 0.0 | 0.0 | 0.0 | 0.0 | 0.0 | 0.0 | 20.0 | 20.0 | 0.0 |
| 10 | 4 | 0.0 | 0.0 | 25.0 | 0.0 | 25.0 | 0.0 | 0.0 | 0.0 | 0.0 | 0.0 | 0.0 | 25.0 | 0.0 | 0.0 | 0.0 | 0.0 |

(c). Andes

| Sr. no | Size | ATP6 | ATP8 | CO1 | CO2 | CO3 | CYB | ND1 | ND2 | ND3 | ND4 | ND4L | ND5 | ND6 | 12s | 16s | trna |
| --- | --- | --- | --- | --- | --- | --- | --- | --- | --- | --- | --- | --- | --- | --- | --- | --- | --- |
| 1 | 567 | 4.6 | 2.3 | 7.9 | 3.5 | 5.3 | 8.1 | 5.5 | 5.5 | 1.8 | 7.4 | 1.2 | 10.1 | 3.2 | 3.0 | 3.7 | 5.5 |
| 2 | 420 | 6.2 | 1.0 | 7.9 | 4.3 | 3.8 | 6.4 | 6.9 | 5.7 | 1.7 | 7.6 | 1.7 | 11.4 | 2.6 | 2.4 | 3.8 | 5.2 |
| 3 | 81 | 7.4 | 0.0 | 6.2 | 6.2 | 6.2 | 7.4 | 4.9 | 6.2 | 2.5 | 11.1 | 4.9 | 11.1 | 4.9 | 1.2 | 6.2 | 3.7 |
| 4 | 57 | 5.3 | 1.8 | 12.3 | 3.5 | 7.0 | 5.3 | 7.0 | 7.0 | 0.0 | 7.0 | 0.0 | 15.8 | 0.0 | 3.5 | 5.3 | 7.0 |
| 5 | 51 | 0.0 | 2.0 | 7.8 | 7.8 | 2.0 | 5.9 | 5.9 | 5.9 | 3.9 | 9.8 | 0.0 | 13.7 | 2.0 | 3.9 | 11.8 | 7.8 |
| 6 | 8 | 0.0 | 0.0 | 12.5 | 0.0 | 0.0 | 25.0 | 12.5 | 12.5 | 12.5 | 0.0 | 0.0 | 0.0 | 0.0 | 0.0 | 0.0 | 12.5 |
| 7 | 8 | 12.5 | 12.5 | 0.0 | 12.5 | 0.0 | 25.0 | 12.5 | 0.0 | 0.0 | 0.0 | 12.5 | 0.0 | 0.0 | 0.0 | 0.0 | 12.5 |
